# Prevalent endosymbiont zonation shapes the depth distributions of scleractinian coral species

**DOI:** 10.1098/rsos.140297

**Published:** 2015-02-11

**Authors:** Pim Bongaerts, Margaux Carmichael, Kyra B. Hay, Linda Tonk, Pedro R. Frade, Ove Hoegh-Guldberg

**Affiliations:** 1 Global Change Institute, The University of Queensland, St Lucia, Queensland 4072, Australia; 2 ARC Centre of Excellence for Coral Reef Studies, The University of Queensland, St Lucia, Queensland 4072, Australia; 3 CARMABI, Piscaderabaai z/n, PO Box 2090, Willemstad, Curaçao; 4 Department of Limnology and Bio-Oceanography, Division of Marine Biology, University of Vienna, Althanstrasse 14, Vienna 1090, Austria

**Keywords:** coral reef, depth distribution, symbiont zonation, mesophotic, *Symbiodinium*

## Abstract

Bathymetric distributions of photosynthetic marine invertebrate species are relatively well studied, however the importance of symbiont zonation (i.e. hosting of distinct algal endosymbiont communities over depth) in determining these depth distributions still remains unclear. Here, we assess the prevalence of symbiont zonation in tropical scleractinian corals by genotyping the *Symbiodinium* of the 25 most common species over a large depth range (down to 60 m) on a Caribbean reef. Symbiont depth zonation was found to be common on a reef-wide scale (11 out of 25 coral species), and a dominant feature in species with the widest depth distributions. With regards to reproductive strategy, symbiont zonation was more common in broadcasting species, which also exhibited a higher level of polymorphism in the symbiont zonation (i.e. number of different *Symbiodinium* profiles involved). Species with symbiont zonation exhibited significantly broader depth distributions than those without, highlighting the role of symbiont zonation in shaping the vertical distributions of the coral host. Overall, the results demonstrate that coral reefs can consist of highly structured communities over depth when considering both the coral host and their obligate photosymbionts, which probably has strong implications for the extent of connectivity between shallow and mesophotic habitats.

## 2. Introduction

Tropical scleractinian corals have been documented across a wide bathymetric range (0 to more than 100 m) within the photic zone [1], with coral species exhibiting distinct vertical distributions [2–4]. The strong environmental gradients encountered across this bathymetric range are important drivers in this zonation, particularly factors such as wave action, heterotrophic resource availability and temperature as they can differ greatly at opposite ends of the depth spectrum [4–7]. However, as scleractinian corals depend to a great extent on photosynthesis for their energy requirements due to their obligate symbiosis with dinoflagellates from the genus *Symbiodinium* [8], the exponential decrease in available irradiance probably plays one of the most important roles in determining the vertical distributions of coral species [4]. Coral species exhibit adaptations along this light gradient to optimize light capture by their endosymbionts, ranging from changes in gross colony morphology [9], skeletal morphology [10], to host pigment composition [11]. In addition, coral populations of the same species at opposite ends of their depth range have been found in association with different *Symbiodinium* types (e.g. [6,12–14]) that can be physiologically distinct and adapted to different light conditions [15,16]. Such ‘symbiont zonation’ (i.e. a bathymetric shift in associated *Symbiodinium*) has been observed for a range of different coral species (e.g. [6,12–14]), and probably facilitates distribution of these species across a broad environmental gradient [16]. However, the prevalence of ‘symbiont zonation’ on a reef-wide scale remains unknown, and it is unclear to what extent it underlies the vertical distributions of coral species.

Most assessments of *Symbiodinium* diversity over depth have been focused either on several species (reviewed in [17]), or community-wide but with low numbers of replicate samples to allow for extensive taxon sampling [18–21]. In addition, most *Symbiodinium* studies have been carried out over narrow depth spans, often sampling target species over a subsection of their entire depth distribution (but see [6,7,22–24]), limiting the power of detecting symbiont zonation. While depth zonation in associated *Symbiodinium* has been observed for coral species with both narrow [12,14] and broad [6,7] depth distributions, it remains unclear whether there is a link between the vertical distribution of the coral host and the occurrence of symbiont zonation (e.g. whether symbiont zonation is more common in corals with broader depth distributions). Similarly, endosymbiont zonation has been found in both brooding and broadcasting coral species [17], but it is uncertain whether *Symbiodinium* zonation is more common in either reproductive mode. Offspring from broadcasting corals generally acquire *Symbiodinium* from the water column (horizontal symbiont transmission), whereas most brooding corals are thought to acquire *Symbiodinium* directly from the maternal colony (horizontal symbiont transmission) [25,26]. This difference in endosymbiont acquisition mode could potentially lead to distinct patterns of *Symbiodinium* diversity over depth [27]. However, assessing links between host traits (i.e. depth distribution and reproductive mode) and patterns of *Symbiodinium* over depth requires extensive genotyping across both a wide number of species and a broad bathymetric range.

The diversity of *Symbiodinium* at mesophotic depths remains poorly explored, and as such, the extent of specialization of mesophotic endosymbiont communities remains unclear [27]. Initial studies in the Caribbean identified putative ‘deep-specialist’ *Symbiodinium* types in the genera *Madracis* [6], *Montastraea* [7] and *Agaricia* [24], pointing towards a certain level of endosymbiont specialization at mesophotic depths. By contrast, preliminary studies on the Great Barrier Reef and in Hawai'i have indicated mesophotic corals associate predominantly with *Symbiodinium* types commonly found in shallow water [27,28]. Nonetheless, most studies of *Symbiodinium* diversity have been carried out over relatively shallow depth ranges, and it may well be that specialization only occurs deeper into the mesophotic zone. In terms of coral species distributions [17,29], the upper mesophotic appears to represent a transition zone between the shallow and lower mesophotic [29,30], and a similar pattern may be present for the associated endosymbiont community. However, the extent of specialization of mesophotic *Symbiodinium* communities and the overlap of endosymbiont communities between shallow and mesophotic depths remains largely untested.

The wide bathymetric distribution of certain coral species, and the relatively shallow depth to which certain stressors and disturbances are limited, has led to the hypothesis that deep reefs may act as refugia and provide a source of reproduction for their shallow-water counterparts (reviewed in [17]). However, recent molecular ecological studies have demonstrated that corals with broad depth distributions do not necessarily form a single metapopulation [23,30–34], and the ability of a species to act as a source of reproduction should therefore not be based on species distribution alone (i.e. co-occurrence in shallow and deep habitats). The relative difficulties associated with the development of informative genetic markers [35] and, more recently, genotyping-by-sequencing approaches for cnidarians hosting endosymbionts still hamper assessment of host population structure on an ecosystem-wide scale (i.e. across a wide range of species), and genotyping efforts have therefore been restricted to case studies of particular species. However, several studies have found that host populations that harbour distinct *Symbiodinium* types over depth (e.g. *Seriatopora hystrix*, *Madracis pharensis* and *Montastraea cavernosa*) are genetically divergent [30–33], illustrating that the occurrence of symbiont zonation can be an indication or ‘proxy’ of underlying host genetic structuring. As such, assessment of symbiont zonation across a broad range of species (which can be assessed using a single *Symbiodinium* marker) may be an important first step towards a better understanding of the potential extent of host population structuring over depth and vertical connectivity of coral communities on a reef-wide scale.

Although we have a reasonable understanding of the vertical distribution of invertebrate species on coral reefs, the role of symbiont zonation underlying these distributions remains poorly understood, particularly on an ecosystem-wide scale. Here, we assess *Symbiodinium* diversity over a large depth range (2–60 m) and across 25 coral species to determine: (i) the overall prevalence of symbiont depth zonation and (ii) whether the occurrence of symbiont zonation is linked to reproductive mode and/or depth distribution of the host. *Symbiodinium* identity was established for 16 broadcasting and nine brooding coral species across depth intervals (2, 5, 10, 25, 40, 50 and 60 m) for a total of 1636 coral colonies from the Buoy 0/1 study site in Curaçao, using ITS2/DGGE fingerprinting. Representing one of the largest genotyping efforts carried out on a single reef, this study demonstrates that coral reefs can consist of highly structured communities when taking into account both the coral host and its associated *Symbiodinium*.

## 3. Material and methods

### 3.1 Sample collection

**Figure 1.**
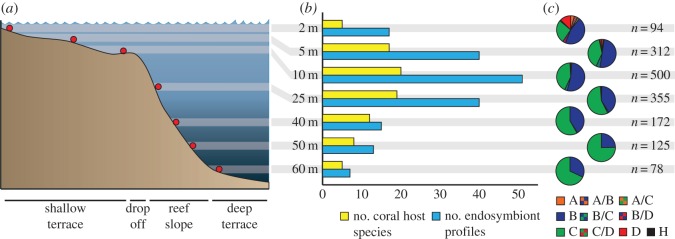
(*a*) Bathymetric profile at sampling location (Buoy 0/1, Curaçao), (*b*) number of coral host species and associated *Symbiodinium* profiles observed across the sampled depth range (2–60 m) and (*c*) prevalence of clades and clade combinations over depth in the *Symbiodinium* profiles of sampled specimens (with numbers of sampled colonies indicated).

Coral samples were collected at the Buoy 0/1 site situated 500 m west of the CARMABI Institute, Curaçao, Southern Caribbean (12^°^07′31 N, 68^°^58′27 W) (figure 1). The study site is characterized by a 50–100 m wide shallow terrace that gently slopes down to 8–12 m, followed by a fore reef slope that shows an abrupt descent down to a 50–60 m deep terrace (figure 1*a*). Colonies were sampled from 25 coral species at this study site, with the majority of samples being collected during the following sampling periods: July–August 2008, November 2009 and the ‘Catlin Seaview Survey’ expedition in March–April 2013. We also included samples from two previously published datasets of the genus *Madracis* (*n*=307; collected between 2004 and 2006) [6,16,32] and *Agaricia* (*n*=332; collected between 2004 and 2009) [24] that were obtained at the exact same study site. Care was taken to avoid resampling of colonies in different sampling years. Small coral fragments (approx. 3 cm^2^) were collected by hammer and chisel at the following depth intervals: 2 m (±1 m), 5 m (±1 m), 10 m (±1 m), 25 m (±2 m), 40 m (±2 m), 50 (±2 m) and 60 m (±2 m) for each species that could be found at that depth. Species identifications were done *in situ*, and later reconfirmed in the laboratory before processing. Coral tissue samples were stored in ethanol or a salt-saturated 20% DMSO/0.5 M EDTA solution at −20^°^C. Total DNA was extracted from the tissue using a Qiagen Plant Mini Kit, MoBio Ultra Clean Soil DNA Kit (following the manufacturer's instructions), or a slightly modified method used for black tiger shrimp [23,36].

### 3.2 Genetic characterization of associated algal endosymbionts (*Symbiodinium*)

The internal transcribed spacer 2 (ITS2) region of the rDNA for *Symbiodinium* was amplified for all samples (*n*=1636) using *Symbiodinium*-specific primers ([18]) as described in Bongaerts *et al.* [31]. To identify the dominant *Symbiodinium* types in each sample, the amplified ITS2 fragments were separated using denaturing gradient gel electrophoresis (DGGE) on a CBScientific System following conditions outlined in Sampayo *et al*. [14]. Running conditions varied for the *Madracis* samples (i.e. 30–70% gradient on Bio-Rad DCode system) included from Frade *et al.* [6], however representative newly acquired *Madracis* samples were run on both systems to allow for cross-comparison between DGGE profiles. Representative, dominant bands of each characteristic profile were excised (usually from several replicate profiles), eluted overnight in dH_2_O, re-amplified and purified (using ExoSAP-IT) prior to sequencing. The re-amplified PCR products were sequenced in both the forward and reverse directions (ABI BigDye Terminator chemistry, Australian Genome Research Facility). Chromatograms were analysed using CodonCode aligner with sequences being aligned with MUSCLE and blasted on GenBank (http://www.ncbi.nlm.nih.gov/BLAST). A haplotype network was created using TCS 1.21 [37], treating gaps as a fifth character state. Network analyses were performed separately for *Symbiodinium* clade B and C because of their high level of divergence. Coral–*Symbiodinium* associations were compared to previously described associations using the GeoSymbio [38] and SD2-GED [39] databases.

The co-dominant sequences in a given profile may represent either intragenomic variants and/or a mix of distinct *Symbiodinium* types, however as this inference can sometimes be subjective we named profiles in this study by their co-dominant ITS2 ‘sequences’ (e.g. C3/C3b/C3nN1) rather than symbiont ‘types’ (which can consist of multiple sequences). Known *Symbiodinium* sequences are indicated by their ITS2 sequence name *sensu* LaJeunesse (e.g. C3d), novel sequences are identified by a nomenclature that specifies the known sequence to which they are most related, followed by a capital N (indicating novel sequence) and an arbitrary number (e.g. C3b.N1) and incomplete sequences (i.e. shorter length) are identified by a nomenclature that specifies the known sequence to which they are most related (based on their partial sequence) followed by a capital U (indicating full sequence remains ‘unknown’) and an arbitrary number (e.g. C3.U1).

### 3.3 Mitochondrial DNA sequence analyses of coral colonies

The coral host mitochondrial *nad5* non-coding region was amplified using primers F18 (5′-GTCCTTACGTCTTTACACCGAC-3′) and R17 (5′-AAAGACCACTCTAAAGCCCGCT-3′) for randomly picked samples from the shallowest and deepest collection depth of each of the coral species in this study (*n*=90) to check for potential cryptic diversity across the sampled bathymetric range (as done for *Madracis* spp. previously by Frade *et al.* [32]). PCR amplifications were performed using conditions described for the *atp6* gene in Bongaerts *et al.* [24], and products were purified, sequenced, aligned and analysed as specified for the algal endosymbionts (*Symbiodinium*). Phylogenetic analyses of sequences were performed using maximum likelihood in MEGA 6 [40] under the delayed transition setting and calculation of bootstrap support values based on 1000 replicates. The best-fit model of molecular evolution was selected by hierarchical Akaike information criterion (AIC) using MEGA 6 [40] with a HKY + G model best describing the *nad5* data under a log likelihood optimality criterion.

### 3.4 Statistical analyses

For host species associating with more than one *Symbiodinium* profile, an (nested) analysis of similarity (ANOSIM) was carried out using a Sorensen resemblance measure in the software package PRIMER v. 6 to test for differences between sampling years (two-way ANOSIM with depth nested within year) and depths (one-way ANOSIM comparing individual depth groups). Data from depths where less than 10 coral colonies were sampled were not included in the ANOSIM. Fisher's exact tests (two-tailed *P*) were used to investigate the relation between reproductive mode and the occurrence of symbiont zonation, and the relation between reproductive mode and the level of polymorphism observed in symbiont zonation (two to three profiles or more than three profiles) using numbers of species as values. A Mann–Whitney *U*-test was used to assess differences in *Symbiodinium* diversity between broadcasting and brooding species. The effect of symbiont zonation and reproductive mode on the depth distribution of the host (determined as the difference between the shallowest and deepest sampling depth) was assessed using two-way ANOVA, with data log(*x* + 10) transformed to meet the assumptions of normality and homoscedasticity.

## 4. Results

### 4.1 *Symbiodinium* diversity and host–symbiont associations

**Figure 2.**
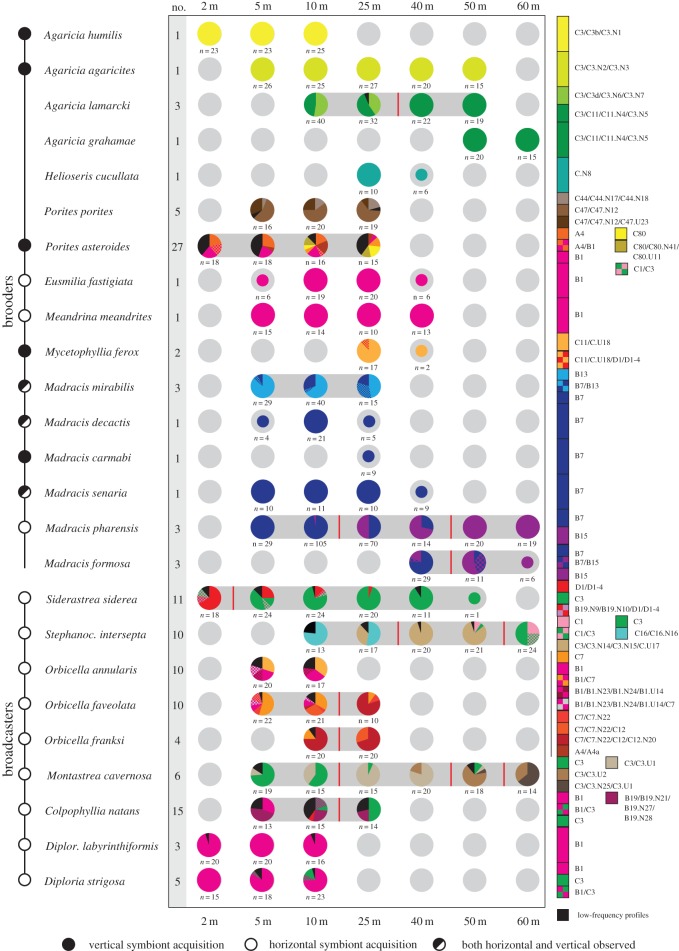
Relative abundances of *Symbiodinium* ITS2 profiles over depth (2–60 m) for 25 scleractinian species on the Buoy 0/1 study site in Curaçao. Pie graphs joined by grey shading indicate significant changes in *Symbiodinium* community over depth (ANOSIM), and red lines indicate ‘break points’ where the *Symbiodinium* community is significantly different at either side of the line. Smaller pie graphs indicate sampling depths where less than 10 colonies were found/sampled (not included in ANOSIM). Coloured circles in front of the species names indicate whether endosymbionts are reportedly acquired vertically, horizontally, facultatively or whether acquisition mode remains unknown (based on [26,34] and P.R.F. & P.B. 2004, personal observations for *Madracis*). Numbers next to species indicate *Symbiodinium* profile diversity (i.e. number of observed profiles).

*Symbiodinium* community profiles were assessed for 1636 coral colonies originating from a single reef, of which 375 originated from mesophotic depths (40–60 m). In total, 93 *Symbiodinium* DGGE profiles were distinguished across the 25 studied coral species (figure 1*b*), based on a total of 120 diagnostic bands. A large amount of profiles only consisted of a single dominant band (41%), however others consisted of either multiple bands from the same *Symbiodinium* clade (24%) or multiple bands belonging to different *Symbiodinium* clades (35%) (figure 2). These diagnostic bands represented 22 known *Symbiodinium* sequences and 39 novel *Symbiodinium* sequences (electronic supplementary material, figure S1) (GenBank accession nos. KP178779–KP178809, KF551185–KF551192), with 22 bands yielding incomplete sequences (after multiple sequencing attempts). Most obtained *Symbiodinium* sequences belonged to either clade C (61%) or clade B (32%), with some belonging to the A, D and H clades which were found exclusively in shallow habitats (less than 3% each) (figure 1*c*).

Several of the observed *Symbiodinium* ITS2 sequences in this study have been observed previously for the same species in other Caribbean locations, such as B7 and B13 in *Madracis mirabilis*, B7 in *Madracis pharensis*, D1a in *Colpophyllia natans*, C3 in *Diploria strigosa* and C12 in *Orbicella faveolata* [38,39]. However, for many species we observed sequences (in both shallow and deep water) that have not been previously identified in association with those species, ranging from closely related variants (e.g. C16.N16 in *Stephanocoenia intersepta*) to distinct clades (e.g. D1a in *Mycetophyllia ferox*) (figure 2). Coral species with a brooding reproductive mode harboured significantly lower numbers of *Symbiodinium* profiles than species with a broadcasting reproductive mode (Mann–Whitney *U*-test, *U*=13, *Z*=−3.4, *p*<0.001). An exception was *Porites astreoides* that hosted a large number of *Symbiodinium* profiles, containing many novel ITS2 sequences. Broadcast-spawning species harboured higher symbiont diversity and many ITS2 sequence variants were identified that were quite divergent from types previously described for those species (e.g. C7 and C16 in *Siderastrea siderea*) (figure 2). Additionally, broadcasting species frequently harboured symbionts from at least two different clades, whereas this was uncommon in brooding species.

### 4.2 Coral host identity

We sequenced the *nad5* region for a subset of individuals (*n*=90) as an initial verification to assess whether shallow and deep populations of collected coral species indeed represent the same ‘species’ (i.e. are not genetically divergent for a conserved DNA region). Lengths of the sequenced *nad5* region varied between 621 and 911 bp depending on whether the coral species belonged to, respectively, the robust or complex phylogenetic group, and sequences were aligned separately based on these groupings (GenBank accession nos. KP178822–KP178861, KP178862–KP178900). Most species (15 out of 19) consisted of a single haplotype per species (with no difference between shallow and deep individuals), and the three species consisting of multiple haplotypes (*C. natans*, *S. intersepta* and *Agaricia lamarcki*) showed no clear partitioning between shallow and deep individuals (i.e. haplotypes were shared) (electronic supplementary material, figure S2). The *nad5* marker was too conserved to distinguish between Faviidae (e.g. *Colpophyllia* and *Diploria*) and members of the *Orbicella* species complex (*O. annularis*, *O. faveolata* and *O. franksi*). Divergence between *Madracis* species was assessed in a previous study 32 that reported genetic divergence between shallow and deep populations of *M. pharensis* at this sampling location (based on the *nad5* marker and two nuclear markers).

### 4.3 Prevalence of symbiont zonation

Depth distributions of individual coral species varied, with absence of *Symbiodinium* data for a species at a particular sampling depth meaning that the species was not observed at that depth during the more than 75 collection dives (conducted to collect all the specimens) on the study site (figure 2). Eleven species with varying depth distributions (Δ depth = 10–55 m) exhibited significant differences in *Symbiodinium* community between sampling depths (i.e. symbiont zonation) (figure 2; electronic supplementary material, table S1). In four cases this consisted of a shift between only two and three *Symbiodinium* profiles (*A. lamarcki, M. mirabilis*, *M. pharensis* and *M. formosa*), and in the other cases consisted of a more complex shift (i.e. involving more than three *Symbiodinium* profiles). By contrast, eight species associated with a single discernable *Symbiodinium* profile across their entire depth distribution (Δ depth = 0–45 m) and five species associated with several different *Symbiodinium* profiles but with no significant differences between depth groups (figure 2). One species (*Madracis carmabi*) was only found at one sampling depth, but hosted a singled *Symbiodinium* profile. No significant differences were found between sample groups collected during different fieldwork periods (two-way ANOSIM with depth nested within year) (electronic supplementary material, table S1).

**Figure 3.**
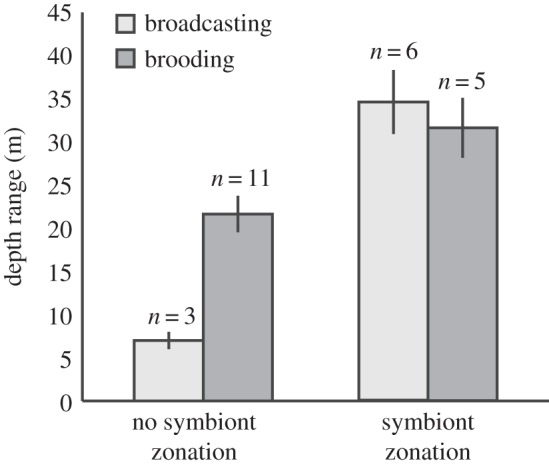
Mean depth distribution ranges for broadcasting and brooding species with and without symbiont zonation. Error bars indicate s.e.m.

Four out of the five species with the largest depth distributions (Δ depth = 40–55 m; *M. pharensis*, *S. siderea*, *S. intersepta* and *M. cavernosa*) exhibited significant symbiont zonation (with *Agaricia agaricites* being the exception), whereas only one of the nine species (*O. franksi*) with the smallest depth distributions (Δ depth ≤ 15 m) exhibited symbiont zonation (figure 2). With regards to reproductive strategy, only five out of 16 brooding coral species exhibited symbiont zonation, while for broadcast-spawning species more than half did (six out of nine), however there was no significant association (Fisher's exact test, *p*>0.05) between reproductive strategy and the occurrence of *Symbiodinium* zonation. Symbiont zonation in all broadcasting coral species was highly polymorphic (i.e. involved more than three *Symbiodinium* profiles), whereas in all brooding species the zonation was ‘simple’ (i.e. involved two to three *Symbiodinium* profiles), with the exception of *P. astreoides*, and this association was significant (Fisher's exact test, *p*<0.05). Species that exhibited symbiont zonation had significantly larger depth distributions (two-way ANOVA, *F*_1,21_=10.0, *p*<0.005), whereas there was no significant effect of reproductive mode or interaction between reproductive mode and symbiont zonation (figure 3) (electronic supplementary material, table S2).

### 4.4 Mesophotic *Symbiodinium* diversity

Compared with shallow depths (5–25 m), the diversity of scleractinian corals and associated *Symbiodinium* diversity (both in terms of absolute numbers and numbers of profiles per species) at mesophotic depths (40–60 m) was substantially lower (figure 1*b*). Depth-generalist species that occurred down to 40–50 m depth mostly harboured the same *Symbiodinium* profile as in shallower water (10–25 m), however depth-generalist species that extend down to 60 m depth (i.e. *S. intersepta*, *M. cavernosa* and *M. pharensis*) all exhibited distinct *Symbiodinium* profiles at their deeper extremes (figure 2). Nonetheless, the common symbiont profiles associated with *S. intersepta* at 60 m represent *Symbiodinium* types (C1 and C3) that commonly occur on shallow reefs (but not in this species at the study location). Depth-‘breakpoints’ were assessed by evaluating pairwise differences between depths for each species (electronic supplementary material, table S1), with ‘breakpoints’ indicating where *Symbiodinium* communities from all depths above and below are significantly different from each other (e.g. for *A. lamarcki* the ‘breakpoint’ is located between 25 and 40 m, as the *Symbiodium* community at 10 m=25 m≠40 m=50 m). The depth at which these ‘breakpoints’ occurred varied between species, however they were most frequently observed between 10 and 25 m (*n*=6), with only one breakpoint observed at shallower depths (i.e. between 2 and 5 m). The remainder occurred at the intersection of the mesophotic zone (between 25 and 40 m; *n*=2) or within the mesophotic zone (between 40 and 50 m or 50 and 60 m; *n*=5).

## 5. Discussion

Depth zonation in associated *Symbiodinium* has been demonstrated for individual scleractinian coral species and genera, with populations hosting distinct endosymbionts at different ends of the depth spectrum (e.g. [12,13,16,41,42]). Here, we demonstrate through extensive genotyping that such symbiont zonation is in fact common among scleractinian corals, with nearly half of the studied species showing significant changes in their *Symbiodinium* community over depth (figure 2). In addition, we found that species exhibiting symbiont zonation have significantly wider depth distributions, which is particularly apparent in broadcasting species (figure 3). We conclude that endosymbiont zonation plays an important role in determining the bathymetric distribution of Caribbean corals, with its overall prevalence leading to highly structured coral host–endosymbiont communities over depth.

Overall symbiont diversity was found to be high, corroborating previous studies in other Caribbean regions [18,21], with only a quarter of the obtained sequences representing known types that have been observed in other parts of the Caribbean. It also corroborates the dominance of clade B and C in this region, with the occurrence of other clades (A, D and H) being restricted almost exclusively to shallow water (10 m or less) [18,21] (figure 1*c*). The lower *Symbiodinium* diversity at mesophotic depths (40–60 m) is in line with previous observations of decreasing diversity over smaller depth ranges in the Caribbean [18,21]. Many new host–*Symbiodinium* associations were observed, with most coral species harbouring profiles containing *Symbiodinium* sequences (ranging from closely related variants to distinct clades) that have not been previously identified in association with those species (figure 2). Observing novel host–*Symbiodinium* associations is common when surveying new geographical regions [43], mesophotic depths [27] or when increasing sample sizes [14], and demonstrates the importance of expanded surveying to broaden our understanding of specificity and diversity in the coral–algal symbiosis. The many novel associations (and ITS2 sequence types) observed here highlight the extensive diversity underlying the geographical and ecological distribution of Caribbean corals.

Symbiont zonation was observed in four out of five coral species with the widest depth distributions, indicating that a change in *Symbiodinium* over depth is a common trait associated with ‘depth-generalist’ coral species. The association with different endosymbionts over depth probably enables these species to thrive under the different environmental conditions encountered along their broad depth distribution ranges [16], through adaptation to particular light [15], temperature [44,45] or potentially other environmental conditions such as light spectrum or nutrient availability [16]. Nonetheless, the fact that *Agaricia agaricites* associated with a single *Symbiodinium* profile along its entire bathymetric distribution (5–50 m) (figure 2) demonstrates that symbiont zonation is not an obligate strategy for depth-generalist species [24]. A similar lack of symbiont zonation has been observed for the Indo-Pacific coral species *Pachyseris speciosa*, which hosted predominantly a single *Symbiodinium* profile despite its broad depth distribution in northwest Australia (10–60 m) [22]. While the observed patterns point towards symbiont zonation being more common in species with large depth distributions, symbiont zonation was also observed over very small depth ranges (e.g. in *Orbicella franski*: Δ depth = 15 m), which is not entirely surprising as certain environmental gradients (e.g. incident irradiance, ultraviolet light and wave action) are most pronounced across shallow depth ranges. Given the inability of ITS2-DGGE to identify *Symbiodinium* at background levels [46] and the fact that only dominant bands are used for profile assignment, assessment of changes in associated *Symbiodinium* diversity over depth remains conservative as it ignores subtle changes in relative *Symbiodinium* abundances. As such, it could be that the frequency of symbiont zonation remains underestimated, however this would not affect the conclusion that *Symbiodinium* zonation is common on a reef-wide scale.

Clear differences were observed between reproductive strategies in the diversity of associated *Symbiodinium*, with broadcasters always associating with multiple *Symbiodinium* profiles (sometimes representing different clades) and with patterns of symbiont zonation always involving at least four (but often more) different *Symbiodinium* profiles. By contrast, brooders often associated with a single *Symbiodinium* profile, and when exhibiting symbiont zonation this usually only involved two to three distinct profiles representing a single clade (with *P. astreoides* as exception). At first, these differences seem to be associated with the different symbiont acquisition modes (respectively, horizontal and vertical) commonly associated with broadcasting and brooding species [25]. Indeed, the broadcasting species studied here reportedly acquire their *Symbiodinium* horizontally [34,47] (figure 2), and the higher *Symbiodinium* diversity and prevalence of symbiont zonation may be facilitated by the ‘open nature’ of the coral–algal symbiosis representative of this acquisition mode [48–50]. The many novel associations that were observed for these species may be a further consequence of this potential ‘flexibility’, and highlights the potential role this acquisition mode has played in the ecological and geographical range extensions of these coral species. By contrast, many horizontally transmitting species in the Indo-Pacific appear to associate with relatively few *Symbiodinium* types [20,51,52], which in part may be a consequence of the overall lower *Symbiodinium* diversity in this region compared with the Caribbean [19].

Brooding species appear to exhibit a variety of *Symbiodinium* acquisition strategies, including vertical (e.g. *Agaricia humilis*), horizontal (e.g. *Eusmilia fastigiata*) and facultative symbiont transmission (e.g. *Madracis mirabilis*) [47] (figure 2). In addition, a recent study reports that *Symbiodinium* can sometimes be acquired through both horizontal and vertical symbiont acquisition (e.g. *Stylophora pistillata* [50]). Thus, while the transmission mode remains unclear for some of the brooding species, the observed pattern is that of low diversity on a localized scale, in line with the relatively ‘closed nature’ of a vertical symbiont acquisition mode. In a vertical symbiont acquisition mode, different *Symbiodinium* associations are thought to arise through (co-)evolutionary processes and/or (relatively rare) host–symbiont recombination events [24,51] that can become fixed through ecological selection or geographical isolation. Such processes are probably responsible for the novel associations observed for these brooding species, and more broadly for geographical differences in *Symbiodinium* diversity of brooding species, rather than merely reflecting symbiotic ‘flexibility’. Similarly, on the Great Barrier Reef, where initially diverse associations where described for several vertically transmitting pocilloporid species (e.g. [14,52]), it later became apparent that these ‘species’ in fact comprise multiple lineages/species with specific endosymbiont associations (e.g. [31,53]). Nonetheless, while *Symbiodinium* diversity associated with individual brooding species is often low on a localized scale, the overall diversity can in fact be high due to biogeographic differences, with higher levels of diversity compared with horizontally transmitting species [54]. The brooding species *Porites astreoides* represents an exception with its extremely high local *Symbiodinium* diversity and indicates that *Symbiodinium* acquisition in this brooding species may not be limited to only maternal acquisition [55], and potentially represents a more ‘open’ symbiosis.

The associated endosymbionts at the lower reaches of this study (50–60 m) appear to represent a specialized community, consisting mostly of *Symbiodinium* that are not observed in shallow water (except, for example, those associated with *A. agaricites*) (figure 2), which is in contrast with preliminary observations from the Indo-Pacific region [27,28]. For some species (e.g. *Stephanocoenia intersepta* and *Montastraea cavernosa*), the symbiont community was distinct even between different depths within the mesophotic zone, which has been observed previously for *M. cavernosa* in the Bahamas [7,33]. Interestingly, the deepest *S. intersepta* populations (60 m) associated at our study site with the generalist types *Symbiodinium* C1 and/or C3 that are commonly found in shallow water [56]. The presence of these *Symbiodinium* at mesophotic depths could be interpreted as a lack of a specialized endosymbiont community, however the absence of these types within the shallow *S. intersepta* populations at our study site points towards (local) specialization of this host–*Symbiodinium* association to mesophotic conditions (60 m). As such, ecological implications of ‘depth-specialization’ should therefore be assessed by looking at the combination of symbiotic partners and on a localized scale (i.e. by comparing shallow versus deep assemblages at a single location). Alternatively, it may be that these *Symbiodinium* are physiologically distinct through recent adaptation to different environmental conditions, as adaptive variation has been demonstrated in *Symbiodinium* C1 previously [57]. At upper mesophotic depths (40 m) of the study site, most of the dominant *Symbiodinium* profiles can be found also at shallower depths for the same species. This indicates that there is substantial overlap in *Symbiodinium* associations between the upper reaches of the mesophotic zone and intermediate/shallow depths for depth-generalist coral species, and reinforces the idea of the upper mesophotic as a transition zone between the shallow and lower mesophotic reef [29,30].

Symbiont zonation has been found to be associated with genetic structuring of the coral host in several brooding and a broadcasting species [7,31–34], and as such can be indicative of underlying host genetic differentiation. The patterns of *Symbiodinium* observed here on a reef-wide scale might therefore be reflective of highly structured coral host populations over depth and genetically distinct populations at lower mesophotic depths. On the other hand, there is considerable overlap in host–*Symbiodinium* associations observed between shallow (10–25 m) and upper mesopotic depths (40 m), highlighting the potential connectedness of these habitats. Nonetheless, host genetic differentiation across habitats has also been observed between populations hosting the same *Symbiodinium* type [31,58], and conversely a lack of host differentiation has been observed for species exhibiting symbiont zonation [59]. Our initial screening of host genetic differentiation was aimed at identifying potential cryptic species diversity and did not reveal any gross genetic differences, other than the depth-divergence of *Madracis pharensis* that was reported in Frade *et al.* [32]. However, the targeted mitochondrial region (*nad5*) was too conserved to assess population-level genetic differentiation (and within the robust clade to assess even intrageneric differences), and the presence of host genetic differentiation underlying the *Symbiodinium* zonation remains to be assessed. The prevalence of *Symbiodinium* zonation stresses the importance of such future host-focused studies, in particular in assessing reef-wide patterns of vertical connectivity.

In conclusion, this study demonstrates the prevalence of endosymbiont zonation underlying the vertical distributions of Caribbean scleractinian corals and indicates that *Symbiodinium* zonation appears to play an important role in facilitating the broad depth ranges of depth-generalist species. Future studies should assess whether this pattern is consistent with other geographical regions and whether endosymbiont zonation is also pervasive in other non-scleractinian photosynthetic marine invertebrates. Overall, we clearly need a better understanding of the ecological and evolutionary interactions between coral hosts and their endosymbionts, through high-resolution genetic studies of both symbiotic partners, to further elucidate reef-wide patterns of host–*Symbiodinium* structuring and the interconnectedness of habitats on coral reefs.

## Supplementary Material

Electronic Supplementary Materials 1 - Figures and Tables

## Supplementary Material

Electronic Supplementary Materials 2 - Original data

## Data accessibility

Sequence data have been deposited in GenBank under the following accession nos. KP178779–KP178809, KP178822–KP178900.
