# Supplementary material for: Prevalent endosymbiont zonation shapes the depth distributions of scleractinian coral species
Source: R Soc Open Sci. 2015 Feb 11;2(2):140297. doi: 10.1098/rsos.140297 (PMC4448818; doi:10.1098/rsos.140297)
Supplement: Electronic Supplementary Materials 1 - Figures and Tables [file rsos140297supp1.pdf]

Electronic supplementary material (ESM)

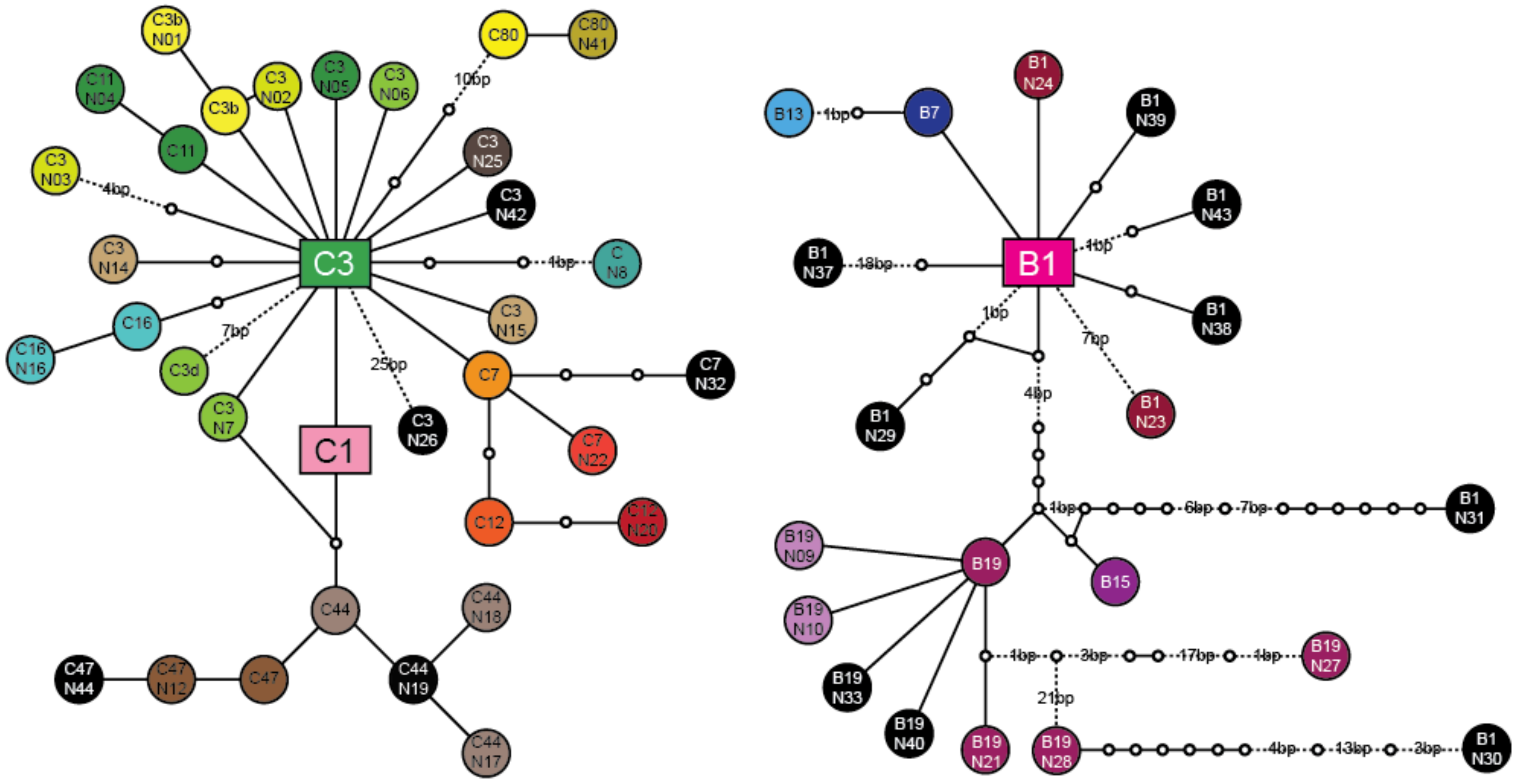

Figure S1. Sequence network of *Symbiodinium* ITS2 types recovered from the DGGE profiles in this study. Colours correspond to those in Figure 3 and indicate in which profile the sequences were observed.

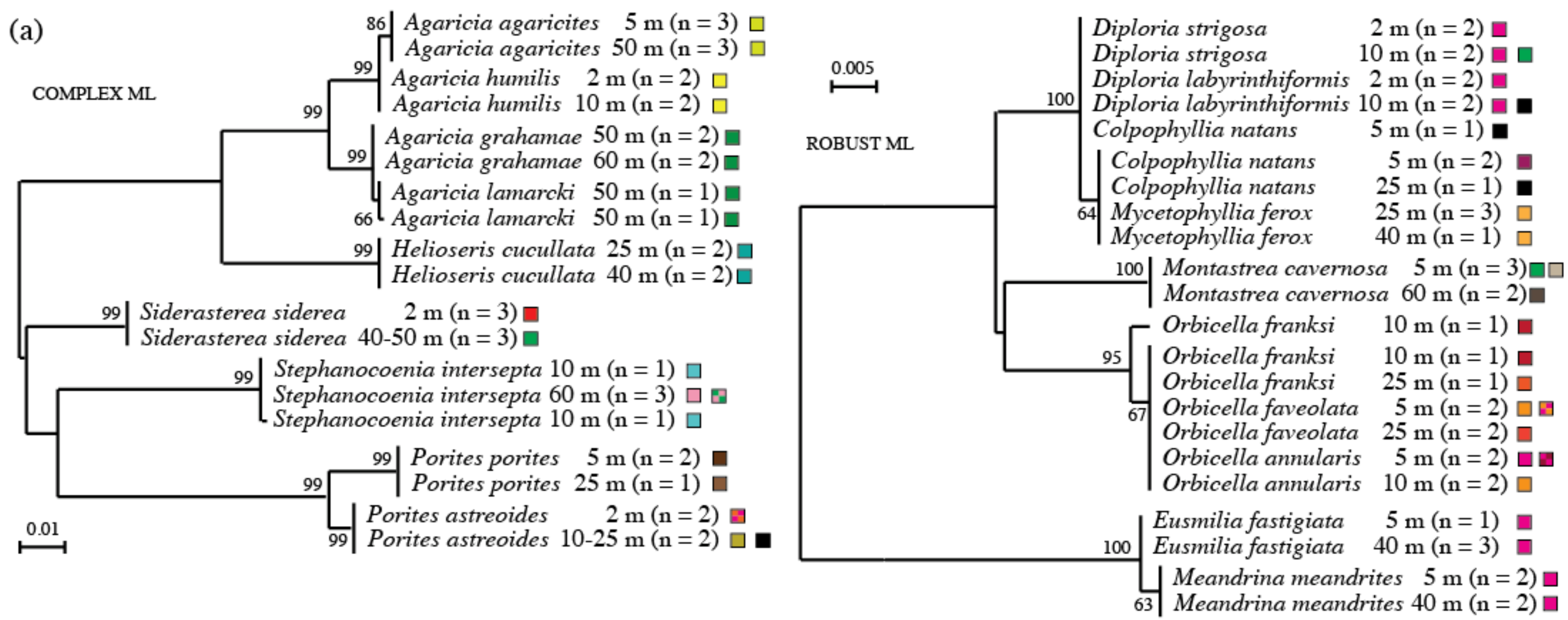

Figure S2. Phylogenetic trees of complex and robust scleractinian coral species based on a mitochondrial (*nad5*) marker. For each species, shallow and deep representative samples were included. Colours indicate the hosted *Symbiodinium* and correspond to the colours in Figure 3.

Table S1. Analyses of similarity (ANOSIM) to test for differences in *Symbiodinium* community between sampling years (Two-Way ANOSIM with depth nested within year) and depth categories (One-Way ANOSIM comparing individual depth groups). Species with either a significant overall depth-effect or significant pairwise depth comparisons are indicated with an asterisk (*).

**Species**	**Year**	**Depth**	
	Two-Way ANOSIM (depth within year)	One-Way ANOSIM - Overall	One-Way ANOSIM – Significant pairwise tests
Agaricia humilis	N.A. (one profile)	N.A. (one profile)	
Agaricia agaricites	N.A. (one profile)	N.A. (one profile)	
*Agaricia lamarcki**	R = -0.135; p = 0.926	R = 0.062; p = 0.009	All significant, except: 10m=25m, 25m=50m & 40m=50m
Agaricia grahamae	N.A. (one profile)	N.A. (one profile)	
Helioseris cucullata	N.A. (one profile / one depth)	N.A. (one profile / one depth)	
Porites porites	N.A. (one year)	R = - 0.011 / p = 0.65	None significant
*Porites astreoides**	R = -0.148; p = 1	R = 0.02; p = 0.078	None significant, except for: 2m<>25m & 5m<>25m
Eusmilia fastigiata	N.A. (one profile)	N.A. (one profile)	
Meandrina meandrites	N.A. (one profile)	N.A. (one profile)	
Mycetophyllia ferox	N.A. (one depth)	N.A. (one depth)	
*Madracis mirabilis**	R = -0.311; p = 0.929	R = 0.041; p = 0.141	None significant, except for: 5m<>25m
Madracis decactis	N.A. (one profile / one depth)	N.A. (one profile / one depth)	
Madracis carmabi	N.A. (one profile / one depth)	N.A. (one profile / one depth)	
Madracis senaria	N.A. (one profile)	N.A. (one profile)	
*Madracis pharensis**	R = -0.004; p = 0.434	R = 0.317; p = 0.0001	All significant, except: 5m=10m, 25m=50m, 25m=60m, 25m=40m, 50m=60m
*Madracis formosa**	N.A. (depth/year confounded)	R = 0.382; p = 0.0002	40m<>50m
*Siderastrea siderea**	R = -0.047; p = 0.595	R = 0.17; p < 0.0001	All significant, except: 5m=40m, 10m=25m, 10m=40m & 25m=40m
*Stephanocoenia intersepta**	R = 0.216; p = 0.179	R = 0.381; p < 0.0001	All significant, except: 10m=25m & 40m=50m
Orbicella annularis	N.A. (one year)	R = -0.014; p = 0.703	None significant
*Orbicella faveolata**	R = -0.296; p = 1	R = 0.144; = 0.0004	All significant
*Orbicella franksi**	N.A. (one year)	R = 0.041; p = 0.047	10<>25m
*Montastraea cavernosa**	R = 0.118; p = 0.195	R = 0.385; p = 0.0001	All significant, except: 5m=10m & 25m=40m
*Colpophyllia natans**	R = 0.111; p = 0.50	R = 0.097; p =0.003	All significant, except: 5m=10m
Diploria labyrinthiformis	N.A. (one year)	R = 0.004; p = 0.289	None significant
Diploria strigosa	N.A. (one year)	R = =0.032; p =0.982	None significant

Table S2. Two-way ANOVA testing the effects of reproductive mode (brooding versus broadcasting) and symbiont zonation (presence versus absence) on the depth distribution ranges of corals (determined as the differential between the shallowest and deepest sampling depth). Data are log + 10 transformed.

Source	df	F	p
Reproductive mode (R)	1, 21	1.5	0.23
Symbiont depth zonation (Z)	1, 21	10.0	< 0.005
R x Z	1, 21	2.2	0.15
